# Editorial: Neuroinflammatory aspects in neurological disorders

**DOI:** 10.3389/fmmed.2025.1557546

**Published:** 2025-02-11

**Authors:** Pinto Alipio, Loidl C. Fabián, Goldstein Jorge

**Affiliations:** ^1^ Laboratorio de Neurofisiopatología, Facultad de Medicina, Instituto de Fisiología y Biofísica “Houssay” (IFIBIO), Universidad de Buenos Aires, Consejo Nacional de Investigaciones Científicas y Técnicas (CONICET), Buenos Aires, Argentina; ^2^ Laboratorio de Neuropatología Experimental, Facultad de Medicina, Instituto de Biología Celular y Neurociencia “Prof. E. De Robertis” (IBCN), CONICET Universidad de Buenos Aires, Buenos Aires, Argentina

**Keywords:** neuroinflammation, neurological disorders & brain damage, Parkinson’s disease, neural mobilization, traumatic brain injury, Guillan-Barré syndrome

**FIGURE 1:**
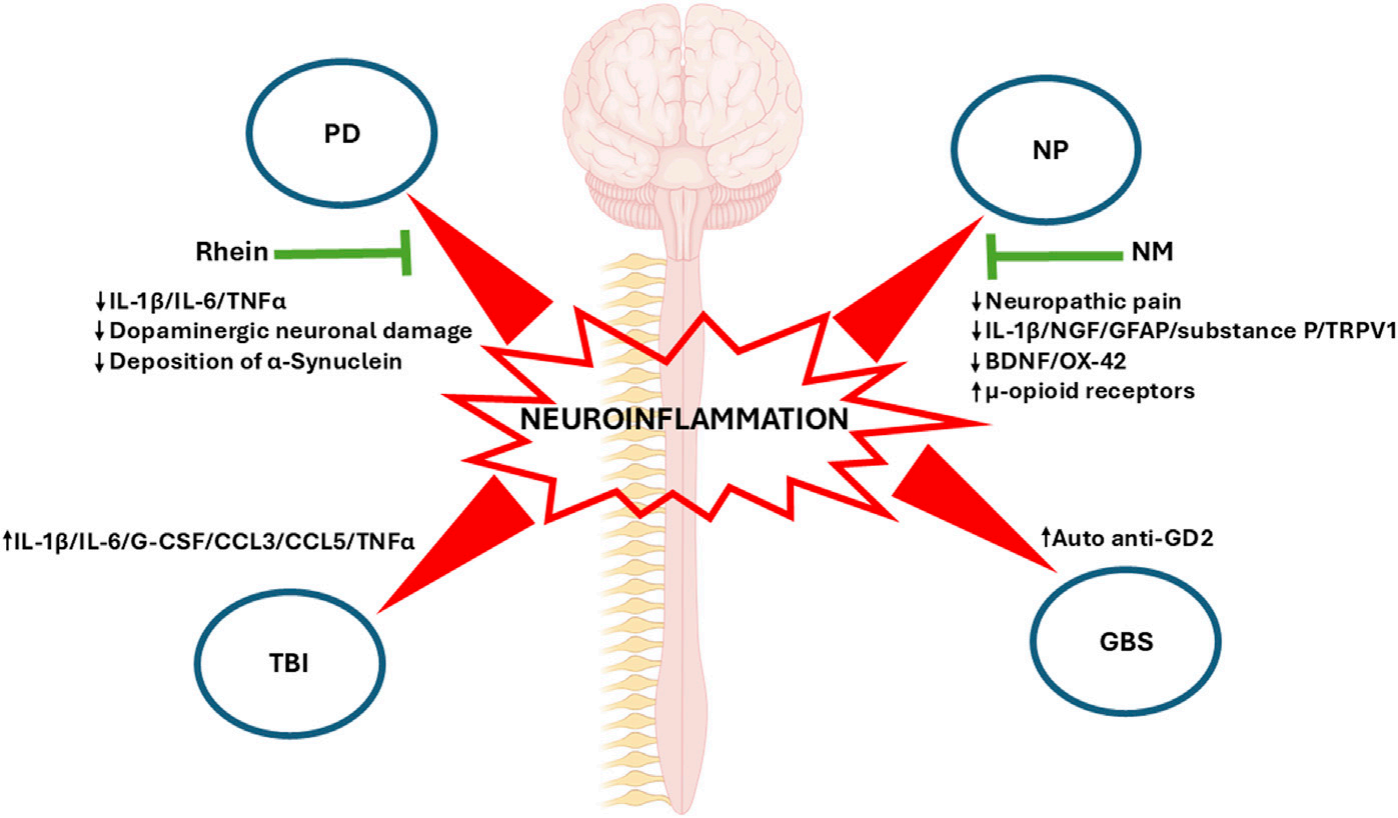
Neuroinflammation in four neurological diseases: The main representative biomolecular markers of each disease presented in this topic are shown. Changes in these markers induced by pharmacological and physiotherapy treatments are highlighted (green arrows). PD, Parkinson’s disease; NP, Neuropathic pain; NM, Neural mobilization; TBI, Traumatic Brain Injury; GBS, Guillain-Barré syndrome; IL-1β, Interleukin-1beta; IL-6, Interleukin 6; TNFα, Tumor Necrosis Factor Alpha; NGF, Nerve Growth Factor; GFAP, Glial Fibrillary Acidic Protein; TRPV1, Transient Receptor Potential Vanilloid 1; BDNF, Brain-derived Neurotrophic Factor; G-CSF, Granulocyte Colony-stimulating Factor; CCL3, C-C Motif Chemokine Ligand 3; CCL5, C-C Motif Chemokine Ligand 5.

Neuroinflammatory mechanisms provide a common basis for a wide range of neurological diseases, which in turn may influence their progression and outcome. A major challenge for medical science, Parkinson’s disease (PD) is characterized by progressive neuronal loss and dysfunction leading to severe cognitive, motor, and behavioral impairments. Neuroinflammation in the central nervous system (CNS) plays a fundamental role in PD progression and continues to spark off intense debate (Cantero-Fortiz and Boada, 2024). Alternatively, neuropathic pain arises from injury or disease of the somatosensory nervous system through immune and glial cell invasion. Given severe neuroinflammation, damage usually outweighs repair in the process of sensitization of somatosensory pain pathways (Scheuren and Calvo, 2024). On the other hand, in traumatic brain injury (TBI), neuroinflammatory mechanisms show a dual component, since it involves glial activation, inflammatory regulator release, and the recruitment of immune cells from the periphery which may produce a secondary injury, and at the same time it is also responsible for the repair of damaged nervous tissue (Zhao et al., 2023). In contrast, Guillain–Barré syndrome (GBS), one of the most common disorders of the peripheral nervous system (PNS), is an example of how neuroinflammatory processes mediated by T cell, macrophage and autoantibodies may produce an acute demyelinating process (Zarobkiewicz et al., 2021). These diseases highlight the central role in which neuroinflammation plays in many neurological disbalances (Figure 1).

The article by Qin et al. addresses the relevance of neuroinflammation in neurodegenerative diseases such as PD and how targeting neuroinflammation can become a valuable therapeutic tool. To this end, Rhein ‒an anthraquinone compound bearing anti-inflammatory properties isolated from plants such aloe vera and used in Chinese medicine‒ was assessed as a therapeutic agent in a mouse experimental model of PD. This molecule succeeded in reducing the canonical inflammatory signaling pathways MAPK/IκB and, consequently, the proinflammatory IL-1β, IL-6 and TNF-α cytokines from substantia nigra and striatum. At the cell level, Rhein reduced dopaminergic neuron damage and the deposition of α-synuclein ‒an extracellular biomarker of neuronal injury‒ and thus attenuated neurological movement disorders (Figure 1).

Salniccia et al. have reviewed neural mobilization (NM), a physiotherapy technique involving passive mobilization of limb nerve structures to restore movement and structural properties in experimental animal models. The authors found that NM alleviated neuropathic pain through opioid system modulation, with regulatory effects of cytokines on the inflammatory and immune systems through modifications of the PNS and CNS. Beneficial effects of NM were also observed on range of motion, limb function, postural control, and muscle strength and endurance. This review unveils the physiological mechanisms underlying NM, which can only be addressed in experimental animal models. For instance, pain models have revealed that NM decreases neural biomarkers such NGF, GFAP, substance P, and TRPV1 but increases u-opioid receptors, associated with higher tolerance to pain, in dorsal root ganglia (Figure 1). In contrast, NGF and myelin protein zero -related to Schwan cell regeneration, nerve recovery, and remyelination‒ increased, and IL-1B decreased in peripheral nerves. Most importantly, GFAP, BDNF, and OX-42, a biomarker of inflammatory microglia, decreased in key pain areas such as the periaqueductal grey and the thalamus following NM. Summing up, physiological studies revealed pain modulation at central and peripheral levels of the endogenous opioid analgesic and immune systems.

A major contributor to mortality and morbidity, TBI is defined as the impairment of brain functionality resulting from consciousness, memory, neurological, or mental state alterations caused by an external force. To et al. studied the relationship between inflammatory markers and white matter integrity in a rat TBI model as well as in clinical TBI cases. Following cortical TBI, rats experienced a time-dependent increase in immunological markers IL1b, IL-6, G-CSF, CCL3, CCL5, and TNF-α (Figure 1), which correlated with white matter preservation, as assessed through the fractional anisotropy (FA) index performed on diffusion tensor imaging (DTI) MRIs to detect traumatic axonal injuries. Similar results were observed in clinical cases, with correlations found in the post-injury period. These results are in contrast with evidence showing that elevated biomarkers in TBI rats and patients are linked with negative outcomes. The findings obtained in this paper warn against ambivalent conclusions which could be drawn from the use of cytokines and chemokines to predict favorable outcomes as compared to more progressive pathology and/or morbidity.

Finally, GBS is an acute demyelinating degenerative neuropathy with inflammatory biases as a consequence of respiratory or digestive viral infection. However, the case presented here by Xu et al. discusses a rare case of GBS, secondary to an ischemic brainstem infarction. An elderly woman hospitalized for weakness and numbness of her left limbs presented walking instability, which coincided with acute paramedian pons infarction, bilateral multiple lacunar cerebral infarctions, and cerebral atherosclerosis, among other outcomes observed in brain imaging. The patient was initially treated for stroke with antiplatelet and lipid lowering therapy, butyphthalide, and edaravone. As symptoms improved, the patient was discharged but had to be hospitalized again due to gradual weakness in the four limbs. Although images showed no bleeding or stroke relapse, symptoms did not improve, and tests targeting a potential infectious origin of limb weakness rendered negative results. The authors argue that cytokines or other molecules damaged by ischemic brain stroke may enter circulation through the injured blood-brain barrier (BBB) or cerebrospinal fluid (CSF), which leads to immunosuppression and, consequently, an increase in auto anti-GD2 antibody to ganglioside, a potential demyelinating autoimmune marker (Figure 1). Accordingly, the patient was diagnosed with GBS and successfully treated with intravenous immunoglobulin.
